# Case of Fractured Ribs, Followed by Extensive Emphysema

**Published:** 1826-09

**Authors:** 

**Affiliations:** the Middlesex Hospital


### INJURIES OF THE THORAX.

### I. *Case of Fractured Ribs, followed by extensive Emphysema.* Treated at the Middlesex Hospital, by Mr. Bell.

J. Boyd, ætatis thirty-eight, was brought to the hospital on the 29th of March, about twelve o'clock at night. The people who brought him stated that he had been found lying in the street, unable to move : he told them that he had been run over by a carriage.

He complained much of his chest, and his breathing was laborious. On pressing the ribs of the left side, several of them seemed to yield; and at each motion of the body a considerable crepitus was felt. In short, it appeared as if nearly all the ribs on the left side had been violently stove in. Although it was evident that several ribs were broken, the exact number could not be ascertained, in consequence of the cellular membrane being already puffed up with air, which had escaped from the lungs, and which was rapidly extending to the other parts of the body. He had great difficulty in breathing, and his pulse was very feeble.

Soon after his admission, the breathing becoming more oppressed, and the pulse rising, an attempt was made to take blood from the arm; but this was rendered difficult by the veins being, as it were, buried and obscured by the emphysematous state of the skin. The quantity of blood thus obtained was small, and afforded but little relief.

The emphysema continued to spread. The loose cellular tissue of the eyelids became so much distended as to close the eyes, and the face was so puffed up as completely to destroy the general outline of the features. The difficulty of respiration increasing, he was bled largely from the temporal artery, until faintness was produced; but, as this in no way relieved the symptoms, Mr. Bell was sent for. He immediately saw the necessity of doing something to relieve the oppression, and suspecting that air was collected in the left cavity of the chest, and encroaching upon the opposite lung, he determined to make an opening into the thorax. He made an incision upon the upper edge of one of the fractured ribs, and, puncturing the pleura, dilated the opening with a bistoury. There was an immediate and forcible rush of air from the chest; insomuch that it almost blew out a candle held before the opening. The breathing was quickly relieved, and even the tumefaction of the face was much lessened.

A few hours after this, the respiration again became laborious, and relief was again obtained by clearing the wound of the coagulated blood with which it was blocked up. It was necessary to repeat the operation at midnight.

Next day (30th), his breathing was easier, but he was troubled with a tickling cough: the expectoration consisted of a glairy mucus, slightly streaked with blood, but there was none of that frothy appearance so common in cases of this kind. A bandage was applied lightly round the thorax in the morning, with a view of steadying the fractured ribs; but the difficulty of breathing was so much increased by it, that it was found necessary to remove it in the afternoon. The bowels were kept open, a saline draught, with antimony and compound tincture of camphor, was administered every six hours; and blood was abstracted as often as the pulse rose sufficiently to warrant it.

On the following morning, the emphysema had diminished, and he continued apparently to improve; but towards afternoon the breathing again became more difficult, and, notwithstanding the free egress afforded to air and extravasated fluids, the oppression continued to increase, and he died in the evening.

*Dissection.*—On examining the body, ten ribs on the left side were discovered to be fractured, three of which projected into the cavity of the chest. Much blood and serum were found in the bag of the pleura on this side; and, on carefully examining the lungs, a laceration was discovered in their texture. The bronchiæ were inflamed, and the cellular structure of the lungs was gorged with blood. A blush of inflammation was general throughout the lining membrane. There were bands of lymph uniting the pleura pulmonalis to the pleura costalis, but these were evidently caused by previous inflammation. The same marks of former pleuritic attacks were found on the right side; which in other respects was natural.

In noticing this case, Mr. Bell observed, that, when emphysema takes place, it is either from the ribs being pressed in upon the lung, or from the lung itself being abraded by the motion of the fractured ribs. In the case under consideration, the ribs were so extensively fractured as to render it almost impossible for the lungs to escape; for three of the fractured ribs projected into the cavity of the thorax.

When the texture of the lung is ruptured, the air escapes into the bag of the pleura during inspiration; and during expiration the air is forced through the wound of the pleura by the side of the fractured ribs, into the common cellular membrane; so that the skin of the whole body is often puffed up in such a degree as to make the patient appear twice his natural size.

Such an appearance is very alarming, both to the patient and to the surgeon; but still there may be little immediate danger, and the respiration may be performed with comparative ease. But, if the air which escapes from the lung does not get a free passage into the general cellular membrane of the body, it will be confined to the cavity of the chest. Under these circumstances, there is great danger; this side of the chest becomes so distended with air, that it not only compresses the wounded lung, but it may so press the mediastinum to the opposite side as to encroach considerably upon the other cavity; whilst the diaphragm is at the same time pressed down, and impeded in its action.

The left lung being wounded, as in the present instance, it becomes collapsed, and the respiration must be carried on by the right lung only; but, if it be impeded by former disease, suffocation then becomes imminent; or if, by the distention of the left cavity with air, the mediastinum be pressed over so as to encroach on the right side, the same danger will be threatened. That this was the condition of the patient in the case before us, was proved by the sudden rush of air from the cavity when punctured; and that the right lung was thus relieved from pressure, and allowed to play freely, was distinctly shown by the immediate relief the patient experienced. Had the surgeon been satisfied with the common practice of general bleeding, and of scarifying the cellular membrane, the patient must have died suffocated; for the scarifications could only have allowed the escape of air from the cellular membrane.

In some cases, the cellular membrane may be but slightly emphysematous, in consequence of the wound of the chest being of a valvular character. Under such circumstances, great oppression of the breathing takes place, and the sufferings of the patient are often of the most distressing kind: his countenance is expressive of extreme anxiety; it exhibits that frantic look which denotes the dread of immediate suffocation.

The presence of air in the general cellular membrane is not in itself a source of danger, as was formerly supposed: indeed, it is remarkable how rapidly it is absorbed. Thus this patient, whom we saw on his admission bloated and swelled to a great size, became afterwards reduced to a state of apparent emaciation.

But still it is natural to inquire, why the patient sunk at the time he did, and after he had been so much relieved? This must be attributed not to the emphysema, but to the inflammation of the lungs and pleura; an almost inevitable occurrence where so extensive an injury has been inflicted in so important a part.

The following cases will serve to illustrate other forms of injury of the thorax.

